# Improving the separation efficient of particles smaller than 2.5 micrometer by combining ultrasonic agglomeration and swirling flow techniques

**DOI:** 10.1371/journal.pone.0239593

**Published:** 2020-09-24

**Authors:** Vladimir N. Khmelev, Andrey V. Shalunov, Viktor A. Nesterov

**Affiliations:** Department of Methods and Tools for Measurement and Automation, Biysk Technological Institute (branch) of the Altay State Technical University, Biysk, Altai Krai, Russian Federation; Tianjin University, CHINA

## Abstract

The method for increasing the separation efficiency of particles smaller than 2.5 micrometers by combined ultrasonic agglomeration and swirling flow technique is proposed in the article. The swirling flow creates areas with an increased concentration of particles on the outer radius of the vortex. The ultrasonic exposure on these areas leads to more efficient agglomeration and the formation of agglomerates of many times larger than the original particles. The resulting agglomerates are easily separated from the gas flow. The design of the agglomerator was developed. The vortex velocity is determined, at which ultrasonic exposure on the swirling flow increases the average particle size *d*_32_ = 2.5 micrometer to 4.5 times. The ultrasonic exposure on a rectilinear flow can increase the particle size no more than 1.6 times for comparison. The proposed method is compared with inertial gas clearing in a cyclone. It was found that the proposed combined method allows increasing the cleaning efficiency from 46% to 85% at ultrasonic exposure on the swirling flow in the agglomerator and cyclone.

## Introduction

The presence and uncontrolled spread of aerosols of various substances in the air have a negative impact on humans, flora and fauna. The existence of a link between the level of atmospheric pollution by fine aerosols of non-biological origin and human mortality rates is proven [1]. Toxicological and epidemiological studies [2, 3] performed in recent years have revealed a correlation between the number of particles with a diameter below 2.5 μm in the air and an increase in cardiovascular and respiratory diseases [4, 5].

The negative impact occurs because the respiratory system introduces fine aerosols into the bloodstream [2, 3] and accumulates toxic material in human organs [6]. The extremely hazardous nature of fine aerosols was confirmed by studies conducted by L. Calderon-Garciduenas, A. Solt, C. Henrıquez-Roldan, and others, who have established a link between the long-term effects of polluted urban air and the sudden death of healthy children and young people [7].

This correlation demonstrates the extreme importance of finding ways to protect people from fine aerosols and developing cleaning systems. However, the existing separators do not efficiently capture particles smaller than 2.5 μm. In addition, they have many related problems: low dust retention capacity (filters) and formation of nitrogen oxides and ozone, which are harmful to the environment and human health (electric filters).

Sound energy offers ways to solve this problem. The application of a high-intensity acoustic field to aerosols causes agglomeration processes, which change the size distribution towards larger particles, which are subsequently easier to capture with a conventional separator [8–13]. This process is called ultrasonic agglomeration.

The phenomenon of coagulation was experimentally discovered by Patterson and Cawood [14] in 1931, and was studied in detail and discussed by Brandt [15], Mednikov [16], Shirokova [17] and Timoshenko [18] and others. The participation of various mechanisms in the process of ultrasonic agglomeration is assumed. It is now generally accepted that orthokinetic, parakinetic and hydrodynamic interactions are the predominant mechanisms. Orthokinetic interaction occurs between two or more suspended particles of different sizes when they are located at a distance approximately equal to the amplitude of displacement of gas molecules in the sound field, and their relative motion is parallel to the direction of vibration. The particles vibrate with different amplitudes and phases due to inertial forces. Such differential motion greatly increases the chance of collision and therefore agglomeration. Parakinetic interaction when two particles of different sizes have their own line of centers not parallel to the ultrasonic field. The transverse interaction of particles is established due to the asymmetry of the Oseen flow field and the curvature of the flow lines. The particles move closer to each other until the collision process occurs in successive periods of gas vibrations.

The hydrodynamic interaction between particles of a monodisperse or polydisperse aerosol under the exposure of an acoustic field is caused by hydrodynamic forces. This forces arising from the mutual distortion of the flow fields around the particles when they are located relatively close to each other along the flow lines.

The distance between particles is a determining factor for the mechanisms of particle interaction as was shown the review. The distance between the particles should not exceed the amplitude of the displacement of gas molecules in the sound field [17] in most cases. This leads to the fact that ultrasonic exposure is effective only for relatively high counting concentrations of suspended particles. However, the particle concentration decreases due to agglomeration [19]. Therefore, the ultrasonic exposure is only effective in the initial stage (when the particle concentration is still sufficient for their convergence and agglomeration). The particle concentration will linearly decrease; therefore, the probability of agglomeration of dispersed particles (and consequently the efficiency of ultrasonic exposure) will decrease in a quadratic dependence over the course of ultrasonic exposure [20, 21].

The various constructions of agglomerators with ultrasonic exposure on suspended dispersed particles [8–11, 22, 23] have been developed. Usually, they are extended volumes and are installed on gas flues in the close proximity of gas cleaning equipment (for example, cyclones [22, 23]). Ultrasonic exposure can be carried out along the direction of gas flow (for circular agglomerators) or perpendicular to the gas flow (for rectangular agglomerators) [8, 9]. Usually, the gas flow mode in the agglomerator is close to laminar and without vortex.

Ultrasonic exposure in agglomerators increases the gas cleaning efficiency. But, it does not solve the main problem of ultrasonic coagulation–low efficiency at a low concentration of suspended dispersed particles. Increasing the time or power of exposure to ultrasonic vibrations does not solve this problem because of the following:

- the typical air flow rates and overall dimensions of modern gas-cleaning equipment do not permit an increase in the time spent by dispersed particles in the ultrasonic field above 0.5 s. In addition, even an unlimited extension of ultrasonic exposure time will not solve the quadratic reduction of the particle agglomeration probability with ultrasonic exposure time [24–27].
- the increase in ultrasonic power is limited by the strength of the emitters (emitters can generate sound level pressure no more than 160 dB without breaking) [28, 29]. Even high-intensity (160 dB) ultrasonic exposure requires more time to aggregate particles smaller than 2.5 μm during a typical residence time in gas cleaning equipment [19, 21, 30].

An auxiliary neutral aerosol (e.g., water) added to increase the probability of collision and ultrasonic agglomeration of particles in most cases is not technically feasible because it leads to corrosion of equipment, formation of sludge, etc. [10, 31].

Also, some articles showing the possibility of using swirling air flows to increase the efficiency of gas cleaning equipment due to the diffusion of particles from the center of the flow to its periphery (the diffusion rate depends on the particle size; larger particles move faster, which increases the chance of their collision with small particles), the effects of Brownian and turbulent coagulation [32]. However, these works are carried out without exposing ultrasonic vibrations on the swirling flow.

Based on the review, the authors of this article offer a method for improving the efficiency of ultrasonic agglomeration of dispersed particles by creating special conditions. Local areas with a high concentration of dispersed particles are formed in the ultrasonic exposure zone. Moreover, in these areas, the decrease in concentration of dispersed particles caused by their agglomeration under the action of ultrasonic vibrations should be compensated by the arrival of new particles.

In practice, conditions for the pre-convergence of particles and formation of areas with an increased particle concentration can be created by creating and detecting the optimal conditions of the swirling flow course with simultaneous ultrasonic exposure. The swirling flow will cause the particles to move to the outer radius under the action of centrifugal forces.

Displacement of particles will result in the formation of areas with an increased concentration.

This is due to the diffusion of particles under the exposure of a centrifugal force away from the center of the vortex. The distance between the particles decreases due to a local increasing of particles concentration in the outer region of the vortex. The efficiency of coagulation mechanisms increases (orthokinetic, parakinetic and hydrodynamic interactions) as the distance between particles becomes smaller. Thus, the main task of the swirling flow is to bring the particles closer to a distance sufficient for them to unite under the exposure of ultrasonic vibrations.

The probability of collision and agglomeration of particles under the action of ultrasonic vibrations will increase with the enlargement of particles to a size sufficient for capture in conventional or modernized separators [31, 33, 34].

The study focuses on the effect of gas consumption and its flow modes (a rectilinear flow or a swirling flow) on the particle agglomeration efficiency and the capture efficiency of the resulting agglomerates. The known dependences of the ultrasonic agglomeration efficiency on the ultrasonic exposure modes (sound pressure level and frequency) [9–12, 34, 35] were taken into account.

### Research stand and measuring means

**Fig 1:**
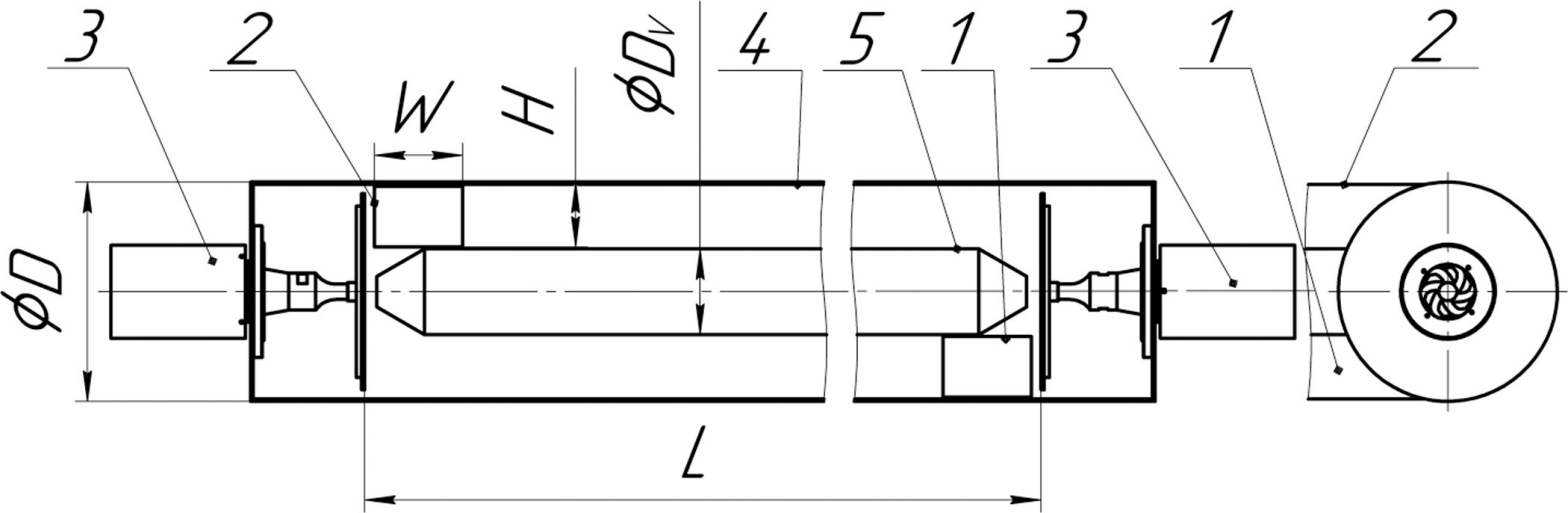
Diagram of the agglomerator with a tangential inlet (swirling flow) 1 –tangential inlet nozzle, 2 –outlet nozzle, 3 –ultrasonic disk radiator, 4 –outer casing of the agglomerator, 5 –displacer, *D*–diameter of the agglomerator, *L*–length of the agglomerator, *H*–height of the inlet and outlet nozzles, *W*–length of the inlet and outlet nozzles.

The research stand (agglomerator) for creating local areas with high concentrations of dispersed particles and their agglomeration under the action of ultrasonic vibrations is designed in the form of a horizontal cylinder (No. 4, Fig 1). The agglomerator has tangential inlet and outlet nozzles (No. 1 and 2). The displacer (No. 5) prevents particles from falling into the axial region, where the centrifugal forces are small [31, 36]. The diameter of the displacer is 1/3 of the diameter of the agglomerator [31, 36].

**Fig 2:**
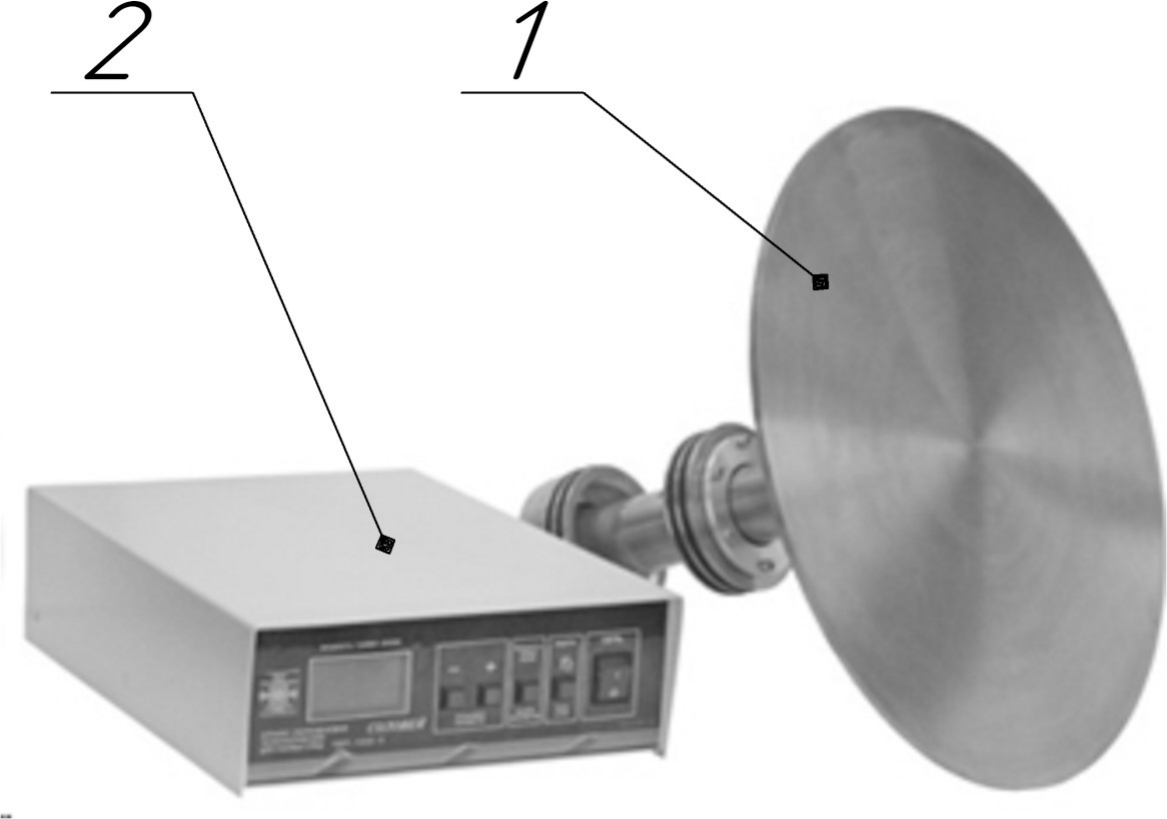
Appearance of the ultrasonic disk radiator. 1 –ultrasonic vibration system with a disk radiator; 2 –electronic generator.

The authors of this article have developed and fabricated two ultrasonic disk radiators (No. 3) to create ultrasonic vibrations with a frequency of 22.5 kHz in the agglomerator. The choice of frequency is due to safety requirements for researchers and the high impact on small-size particles [13, 35]. The principle of operation of the developed radiators and their technical characteristics are discussed in detail in other works by the authors [9, 28, 29]. The ultrasonic disk radiator with an electronic generator is shown in Fig 2 [28, 29], and its technical characteristics are presented in Table 1.

**Table 1:** Technical characteristics of the ultrasonic disk radiator.

Parameter	Value
Radiator diameter, mm	360
Maximum sound pressure level on the acoustic axis, dB, not less than	159
Resonant frequency of the oscillatory system, kHz	22.5

The agglomerator operates as follows. The gas flow containing dispersed particles enters the agglomerator through the tangential inlet nozzle (No. 1). Inside the agglomerator, as a result of the movement along a spiral trajectory, under the action of centrifugal forces, the gas-dispersed flow separates, and the dispersed particles are displaced to the peripheral flow area towards the outer casing of the agglomerator (No. 4). There, the particles are exposed to ultrasound, which results in their coagulation and the formation of agglomerates. Increasing the weight of the agglomerates leads to a further increase in the degree of flow stratification. The gas flow containing the agglomerates of dispersed particles leaves the agglomerator through the outlet nozzle (No. 2).

**Fig 3:**
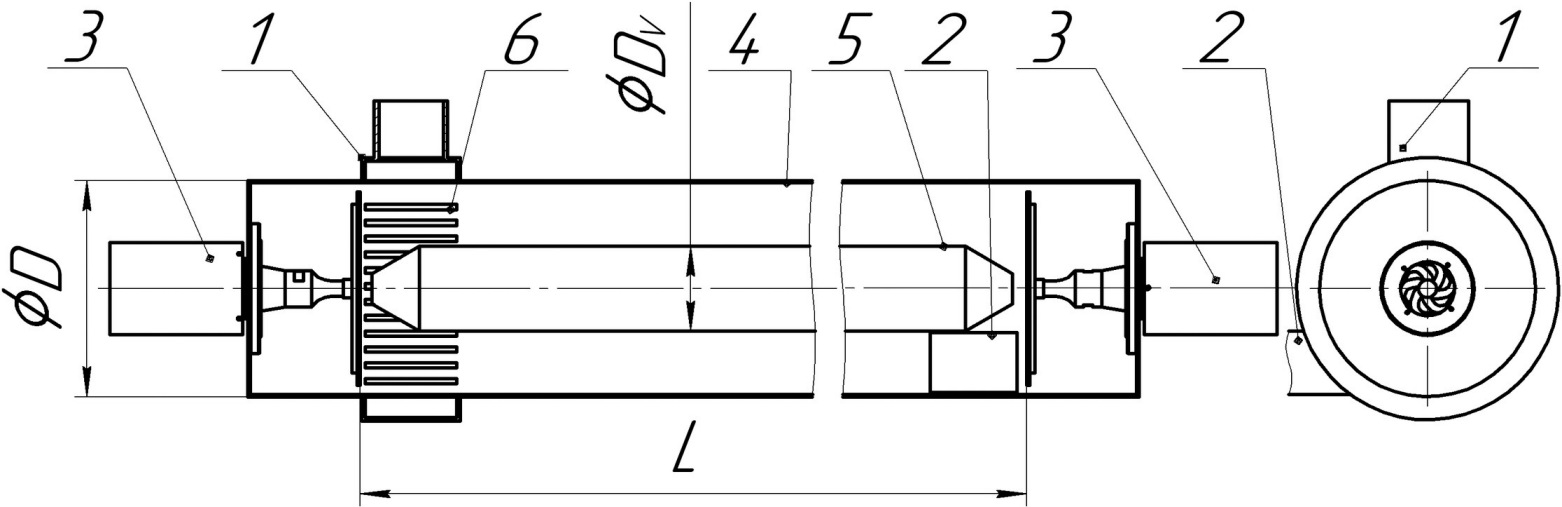
Diagram of the agglomerator with a flow distributor (rectilinear flow). 1 –inlet nozzle with flow distributor, 2 –outlet nozzle, 3 –ultrasonic disk radiator, 4 –outer casing of the agglomerator, 5 –displacer, 6 –slotted openings of flow distributor; *D*–diameter of the agglomerator, *L*–length of the agglomerator, *H*–height of the inlet and outlet nozzles, *W*–length of the inlet and outlet nozzles.

The replaceable inlet nozzle, which rectilinearly directs the gas-dispersed flow, was designed to compare the coagulation efficiency in a swirling flow and a rectilinear flow. The tangential nozzle (No. 1, Fig 1) with a cylindrical part of its case is replaced by a flow distributor (No. 1, Fig 3) to form a rectilinear flow (Fig 3). In this design, the gas-dispersed flow is uniformly supplied through slotted openings (No. 6, Fig 3) of the cylindrical part of the agglomerator outer casing (No. 4), which ensures a rectilinear flow in the volume of the agglomerator.

To ensure maximum efficiency and uniformity of ultrasound exposure, the inner diameter of the agglomerator corresponds to the diameter of the disk radiator (0.36 m). The length of the agglomerator is selected to ensure the time of exposure to ultrasonic vibrations on a particle moving in the gas flow for at least 1 s [24–27].

The gas flow conditions must correspond to a straight-flow cyclone (which also uses centrifugal forces to separate the particles) since suspended particles in the agglomerator are displaced from the center to the periphery of the vortex by centrifugal forces.

The gas consumption is in the range of 500–1500 m^3^/hour for cyclones with diameters of 0.3–0.4 m according to references [37, 38]. The optimal gas flow rate depends not only on the diameter of the cyclone, but also on its purpose and construction. Therefore, an average value *Q* = 1000 m^3^/hour was chosen as the initial gas flow for the proposed cyclone construction with a diameter of 0.36 m. The selected flow rates enable us to calculate the length of the agglomerator using the following formula [24–27]:

$$L=\frac{4QT}{\pi (D^{2}-D_{v}^{2})}$$ (1)

where *Q* is the gas flow rate, m^3^/s; *T* is the exposure time, s; *D* is the diameter of the agglomerator, m; and *D*_*v*_ is the diameter of the displacer, m.

Considering that *D*_*v*_ = *1/3D*, formula (1) is rewritten as follows:

$$L=\frac{4QT}{\pi (D^{2}-\frac{1}{9}D^{2})}=1.43\frac{QT}{D^{2}}$$ (2)

To ensure that the time of ultrasound exposure is at least 1 s, the length of the agglomerator must be at least 3 m. The height of the inlet and outlet tangential nozzles corresponds to:

$$H=\frac{D-D_{v}}{2}=\frac{1}{3}D=0.12\text{m}.$$ (3)

The initial tangential velocity *Vτ* was limited to a value of 15–20 m/s, since an increase in the gas velocity in the cyclone dust collector inlet above 20 m/s leads to increased turbulence and a decrease in the separation efficiency [33].

Considering formula (3), width *W* of the inlet and outlet nozzles at the selected flow rate *Q* = 0.28 m^3^/s (1000 m^3^/h) and tangential speed (20 m/s) is calculated using the following formula:

$$W=\frac{3Q}{V_{\tau }D}=0.12\text{m}.$$ (4)

**Fig 4:**
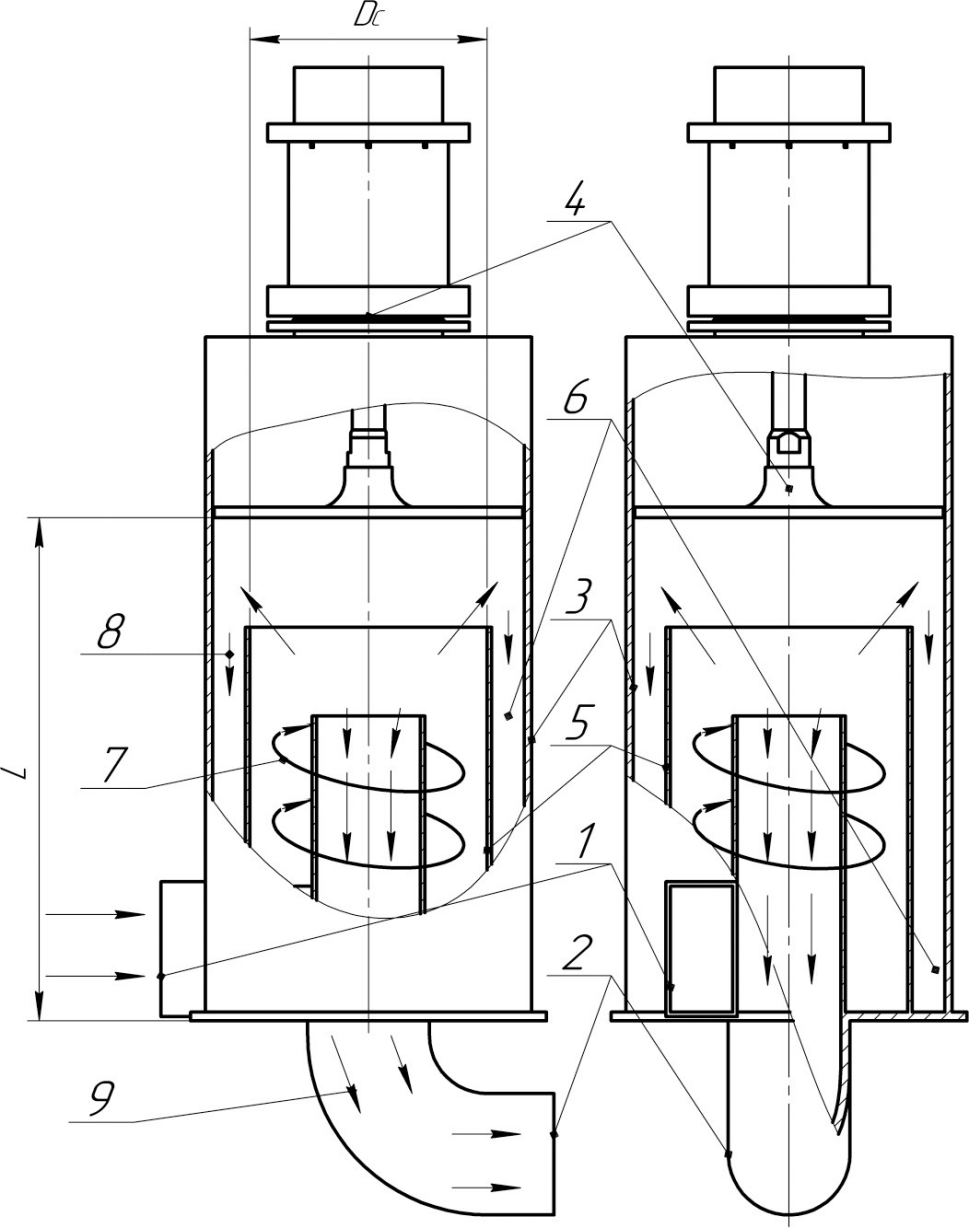
Diagram of the modified cyclone dust collector. 1 –inlet tangential nozzle, 2 –outlet nozzle, 3 –casing, 4 –ultrasonic disk radiator, 5 –separation chamber, 6 –bunker; 7 –direction of gas flow in the separation chamber (upstream), 8 –direction of particles in the bunker, 9 – downstream.

To confirm the effectiveness of the proposed method of cleaning gases from the dispersed particles, the device for forming a swirling flow (the agglomerator) was completed with a known cyclone dust collector [39]. The cyclone dust collector was modified by installing an ultrasonic disk radiator, which was developed by the authors (Fig 4). The cyclone dust collector inlet was connected to the agglomerator outlet.

This type of cyclone dust collector is selected because the motion pattern of dispersed particles in it has the form of a swirling flow. The ultrasonic exposure ensures further consolidation of particles, and the largest agglomerates are removed in that device. Non-removed agglomerates (with a low residual concentration) that have passed to the outlet of the cyclone dust collector with the gas flow can be easily captured by conventional separators, e.g., filters or electric filters.

In the modified cyclone dust collector, the gas flow that contains dispersed particle agglomerates pre-formed in the first stage tangentially flows through the inlet nozzle (No. 1) into the separation chamber (No. 5), where it moves in an upstream spiral (No. 7). Under the effect of a centrifugal force arising from the rotational movement of the flow, the particles move to the outer walls of the separation chamber (No. 5). The dispersed particles are separated from the gas at the transition from the upstream flow (No. 7) to the downstream flow (No. 9) and enter the bunker (No. 6).

The selected size of the cyclone dust collector ensures the required flow rate *Q* = 0.28 m^3^/s (1000 m^3^/h), and it has a separation chamber diameter *D*_*c*_ = 0.3 m and a length *L* = 0.9 m.

**Fig 5:**
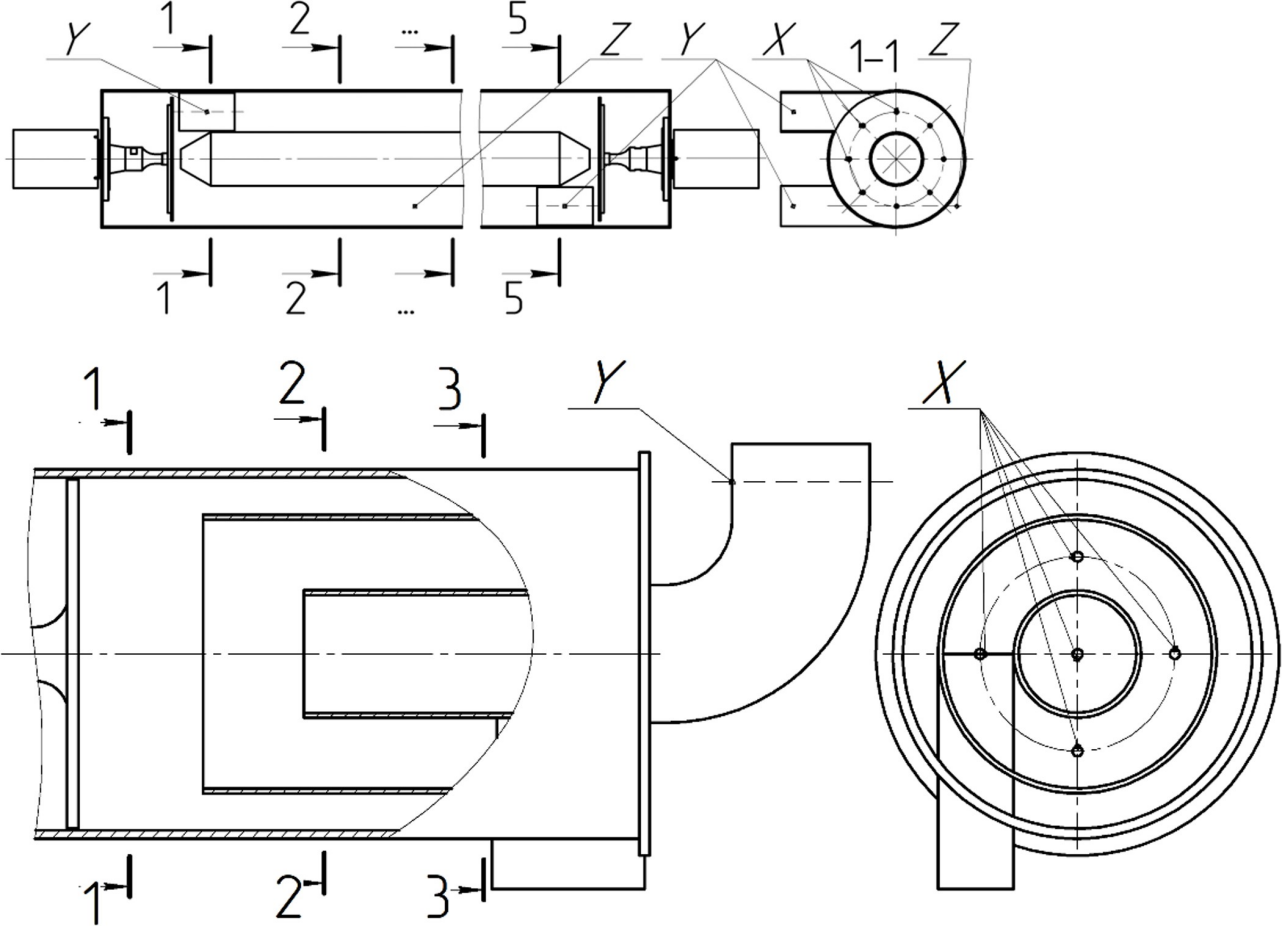
Points of measurement of sound pressure and average diameter of dispersed particles. A–agglomerator; B–cyclone dust collector; Dashed line–laser beam path; X–points of sound pressure measurement in each plane; Y–points of measurement of the average diameter of dispersed particles in a flow; Z–points of measurement of the average diameter of dispersed particles at a zero flow rate.

The sound pressure level generated by the ultrasonic radiators in the internal volume of the agglomerator (Fig 5A) and the cyclone dust collector (Fig 5B) of the research stand were measured before the experimental studies.

Measurements were made at 40 points in the agglomerator and 12 points in the cyclone dust collector. The noise meter CASELLA CEL-633 was used to measure the distribution of the sound pressure level generated by ultrasonic radiators in the agglomerator and cyclone dust collector. The points of sound pressure measurement are shown in Fig 5.

The measurements show that the average sound pressure level was 145 dB (at 22.5 kHz). The deviation from the average value at different measurement points does not exceed 3 dB. Thus, the level of sound pressure is sufficient for the coagulation of dispersed particles and their uniform distribution in the experimental stand [28, 29].

**Fig 6:**
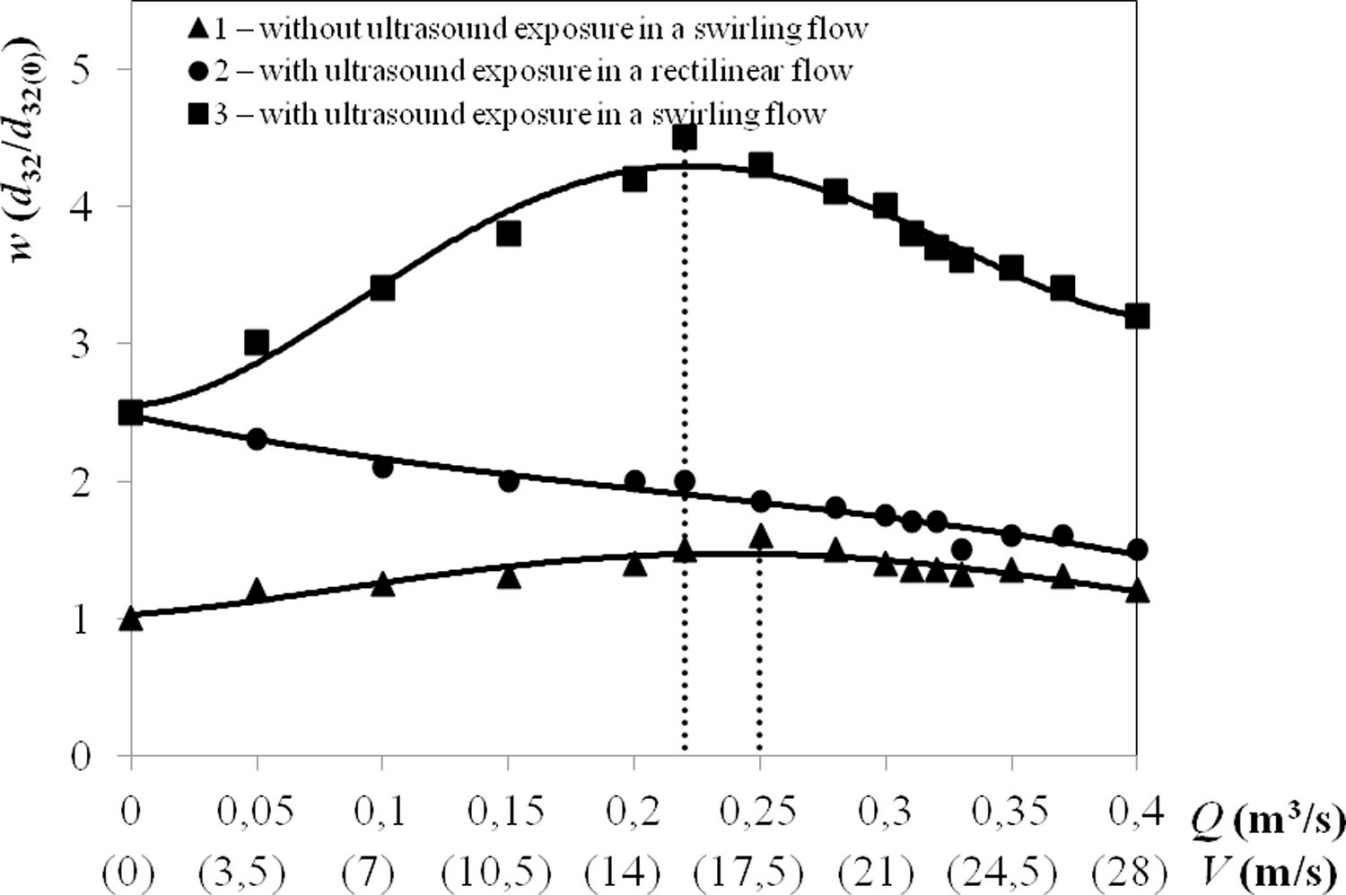
Dependence of the relative enlargement of dispersed particles on the gas flow rate (*V*) at different gas flow modes.

The experimental stand was equipped with the following measuring means of measuring the gas-dispersed flow characteristics:

- Kimo LV-110 thermo-anemometer (Kimo Instruments, France) to measure and monitor the gas velocity and flow rate through the research stand. It was placed in the inlet of the nozzle for measurements;
- laser dispersion meter (LID-2M) (0.1–100 μm particle size) to measure the mean volume-surface diameter (*d*_32_) and concentration of dispersed particles [40–42]. Openings to install emitters and receivers of the LID-2M meter were made in the inlet and outlet nozzles of the research stand (Fig 5A pos. Y). Additional openings were arranged in the centre of the outer casing of the agglomerator (Fig 5A, pos. Z) to determine the average diameter of dispersed particles at zero flow rate (the starting point is shown in Fig 6).

Jetfine T1 CA microtalc was used as dispersed material. True density: 2.69 g/cm^3^; bulk density: 0.8 g/cm^3^ (according to the manufacturer). The dispersed particles were fed by an ejection-type pneumatic atomizer mounted in the inlet nozzle of the agglomerator. A uniformly distributed flow of particles with an average diameter of *d*_32_ = 2.5 μm was formed as shown by measurements made by the LID-2M meter.

## Results and discussion

### Ultrasonic agglomeration efficiency in the swirling flow

The agglomeration efficiency was evaluated by a relative enlargement of the dispersed particles exposed to ultrasonic vibrations in the agglomerator at different gas flow rates and modes (rectilinear or swirling). The relative enlargement of dispersed particles was defined as *w* = *d*_32_*/d*_32(0)_, where *d*_32_ is the average diameter of particles at the agglomerator outlet and *d*_32(0)_ is the average diameter of particles at the agglomerator inlet.

The relative enlargement of particles was used since the main purpose of the agglomerator is to increase the ultrasonic agglomeration efficiency by increasing the concentration of particles in the peripheral region of the vortex flow. The agglomeration efficiency is higher if the larger the diameter of the particles at the outlet of the agglomerator (or the higher the ratio of the diameter of the particles at the outlet of the agglomerator to the initial diameter of the particles). In addition, this parameter does not depend on the initial particle size and allows you to determine how many times the average number of elementary collisions of dispersed particles has increased. The number of elementary acts of collision of dispersed particles can be calculated as the relative enlargement of particles raised to the third power (the formula for the volume of a sphere was used).

Experiments in swirling and rectilinear flows were carried out at gas flow rates of 0.05–0.4 m^3^/sec. The concentration of particles injected at the inlet of the agglomerator was maintained at *N* = 5 g/m^3^ for all gas flow rates.

A motionless gas-dispersed suspended matter (initial points of graphs No. 1, No. 2 and No. 3 in Fig 6) was formed by filling the agglomerator with dispersed particles at a gas flow rate of 0.05 m^3^/s. Thereafter, the gas supply was stopped. The mean diameter of motionless suspended particles was measured using the LID-2M dispersion meter at point Z (Fig 5A) with simultaneous ultrasonic exposure (starting points of graphs No. 2 and No. 3 in Fig 6) and without it (starting point of graph No. 1 in Fig 6). The results are shown in Fig 6.

Having analysed the obtained results, we draw the following conclusions:

1. The creation of a swirling flow without ultrasonic exposure (graph No. 1 in Fig 6) provided enlarged particles at a flow rate *Q* = 0.25–0.3 m^3^/s due to their spontaneous agglomeration of 1.6 times. This result confirms the feasibility of using a swirling flow to locally increase the particle concentration to increase their collision probability.
2. Ultrasonic vibrations provided a relative enlargement of particles by up to 2.5 times (starting point of graphs No. 2 and No. 3 in Fig 6) for motionless gases. The maximum effect of ultrasonic exposure is observed in the first second of the experiment (particles increase in size by up to 2 times). The particle size increased by up to 2.5 times in the next 10 s of the experiment. Further ultrasonic exposure did not result in particle enlargement due to a decrease in particle concentration by 16 times (by coagulation). As a result, the formed agglomerates stop increasing in size and deposit by gravity.

The increase in the rectilinear flow rate (graph No. 2 in Fig 6) reduced the relative enlargement of dispersed particles by up to 1.5 times. The reason is the decreased time spent by the particles in the ultrasonic field, which is not more than T = 0.68 s for the maximum flow rate of *Q* = 0.4 m^3^/s (1440 m^3^/h).

3. The creation of a swirling flow and ultrasonic exposure changed the dependence of the relative enlargement on the gas flow rate (graph No. 3 in Fig 6) and increased the particle size by up to 4.5 times.
4. The maximum increase in the size of dispersed particles in a swirling flow at the ultrasonic exposure was achieved at a gas flow rate of *Q* = 0.22 m^3^/s (800 m^3^/hour). There is an extremum because the tangential velocity of the swirling flow increases and the time spent by dispersed particles in the agglomerator decreases with increasing gas flow.

Thus, these studies enable us to identify the modes and conditions of formation of the dispersed flow with the maximum relative consolidation of particles at ultrasonic exposure.

### Comparison of gas cleaning by combined and inertial methods

In these experiments, a cyclone dust collector (modified by combining it with an ultrasonic radiator) was connected to the output of the agglomerator (Fig 4). The gas cleaning efficiency was determined as follows:

$$\eta =\left(1-\frac{C_{out}}{C_{in}}\right)\cdot 100\%,$$ (5)

where *С*_*in*_ is the mass concentration of particles at the agglomerator inlet, g/m^3^; *С*_*out*_ is the mass concentration at the cyclone dust collector outlet, g/m^3^.

In these experiments, the enlarged particles (agglomerates) were fed from the outlet of the agglomerator to the cyclone dust collector. Further coagulation of the particles under the action of ultrasonic vibrations and their separation from the gas flow under the action of inertia forces were carried out in the cyclone dust collector. The particle concentration at the agglomerator inlet and cyclone dust collector outlet was measured using an LID-2M dispersion laser meter [40–42]. The characteristics of the gas-dispersed flow were as described in the previous section.

**Fig 7:**
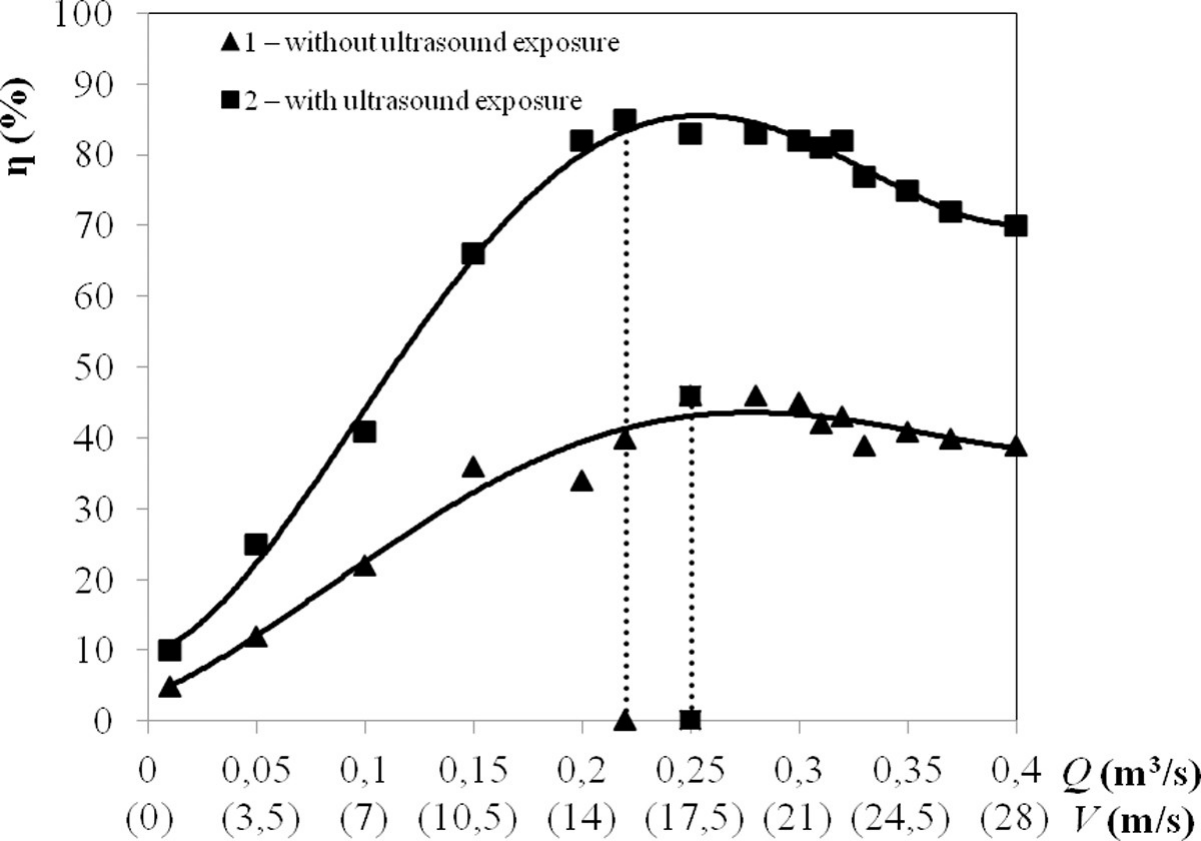
Dependence of the cleaning efficiency on the gas flow rate. 1 –without ultrasonic exposure; 2 –with ultrasonic exposure.

The dependences of the cleaning efficiency on the gas flow rate are presented in Fig 7.

The dependency analysis leads to the following conclusions:

1. With minimum gas flow rates (*Q* = 0.01 m^3^/s), the efficiency of cleaning without ultrasonic exposure (starting point at graph No. 1 in Fig 6) did not exceed 5%. The imposition of ultrasonic vibrations (starting point at graph No. 2 in Fig 6) increased the cleaning efficiency by up to 10% by enlarging the dispersed particles exposed to ultrasonic vibrations.
2. The maximum efficiency of cleaning from particles *d*_32_ = 2.5 μm without ultrasonic exposure reached 46% at a gas flow rate of *Q* = 0.25 m^3^/s (graph No. 1 in Fig 7).

This result corresponds to the available literature data [26, 27] on the effectiveness of gas cleaning equipment for dispersed particles with a size of 4–6 μm. Particles of initial size *d*_32_ = 2.5 μm at a gas flow rate 0.25 m^3^/s were enlarged in the agglomerator by 1.6 times (up to 4 μm) (see Fig 6).

3. With ultrasonic exposure, the maximum cleaning efficiency increased by 39% and reached 85% at a gas flow rate of *Q* = 0.22 m^3^/s due to the preliminary enlargement of particles in the agglomerator (4.5 times for the maximum value on graph No. 2 in Fig 7) and additional enlargement in the cyclone dust collector under exposure to ultrasonic vibrations.

Thus, the obtained results show a 39% increase in gas cleaning efficiency of particles of size *d*_32_ = 2.5 μm due to the ultrasonic exposure of the swirling flow.

**Fig 8:**
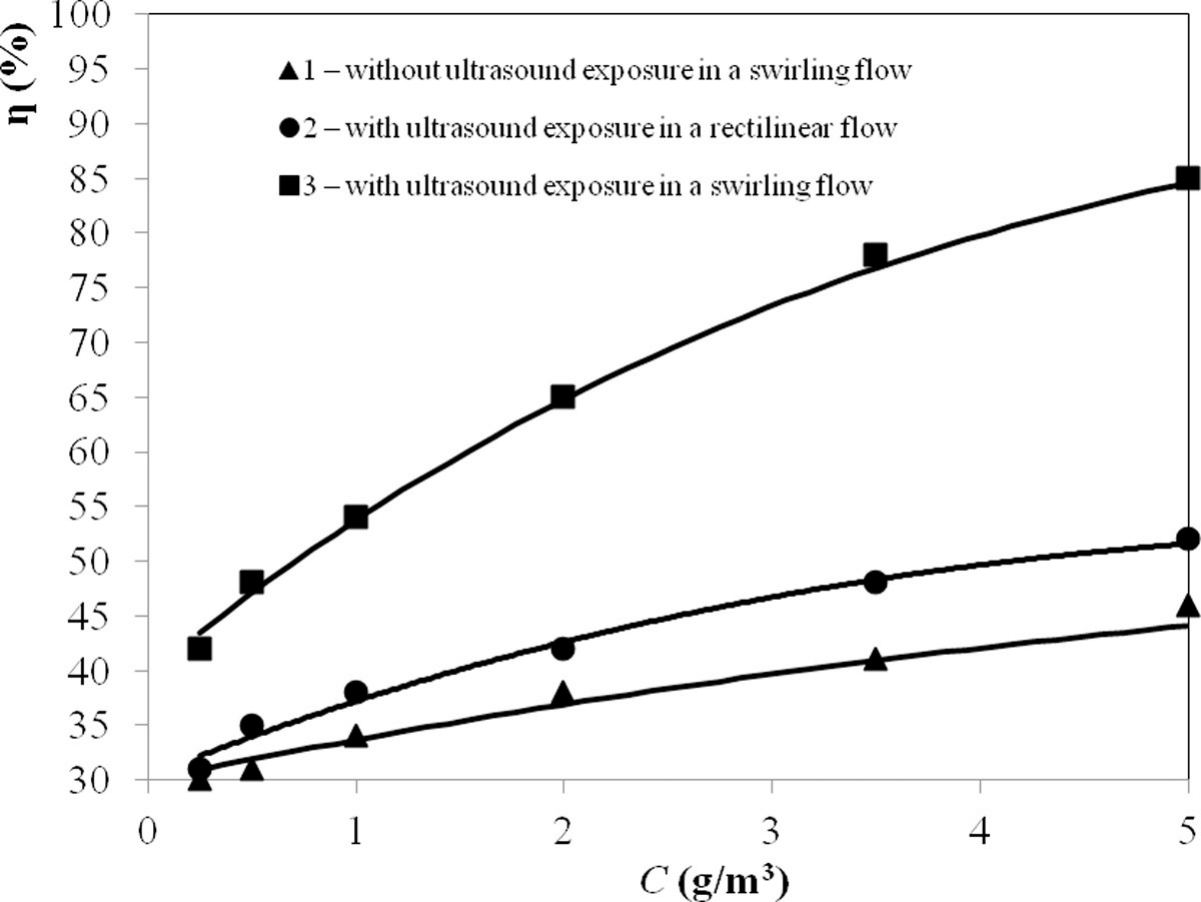
Dependence of the cleaning efficiency on the mass concentration of particles (*d*_32_ = 2.5 μm) at the agglomerator inlet.

Further studies with the identified optimum gas flow rates (*Q* = 0.22 m^3^/s–with ultrasonic exposure; *Q* = 0.25 m^3^/s–without ultrasonic exposure for a comparison of results) aimed to determine the effect of the concentration of dispersed particles on the effectiveness of ultrasonic agglomeration. The results are presented in Fig 8.

Fig 8 shows that the ultrasonic exposure of the swirling flow ensures an increase in the cleaning efficiency from 12% (42% - 30% with a particle concentration of 0.25 g/m^3^) to 39% (85% - 46% with a particle concentration of 5 g/m^3^) compared to a swirling flow without ultrasonic exposure. In addition, the ultrasonic exposure of a rectilinear flow (in the agglomerator) does not significantly increase the gas cleaning efficiency (at a concentration of 5 g/m^3^, the efficiency increases by 6% in comparison with a swirling flow without ultrasonic exposure). This result indicates the effectiveness of the proposed method to increase the efficiency of gas cleaning by creating a swirling flow and its ultrasonic exposure.

To confirm the efficiency at which particles of various sizes are trapped, experimental studies were carried out using microtalcs of various grades with the average particle size ranging from 1 to 17 μm with the identified optimum gas flow rate and initial particle concentration *N* = 5 g/m^3^. The experiments were carried out with four different combinations:

1. Experiment 1: Only the cyclone was used. Control experiment.
2. Experiment 2: The cyclone was connected in series to the agglomerator. The cleaning efficiency without ultrasound was determined.
3. Experiment 3: The cyclone was connected in series to the agglomerator. The ultrasonic exposure was only in the agglomerator. The cleaning efficiency increasing is determined by combining the methods of ultrasonic agglomeration and swirling flow.
4. Experiment 4: The cyclone was connected in series to the agglomerator. The ultrasonic exposure was in the agglomerator and cyclone. Proves the need to use a two-stage gas cleaning system with ultrasonic exposure at each stage.

**Fig 9:**
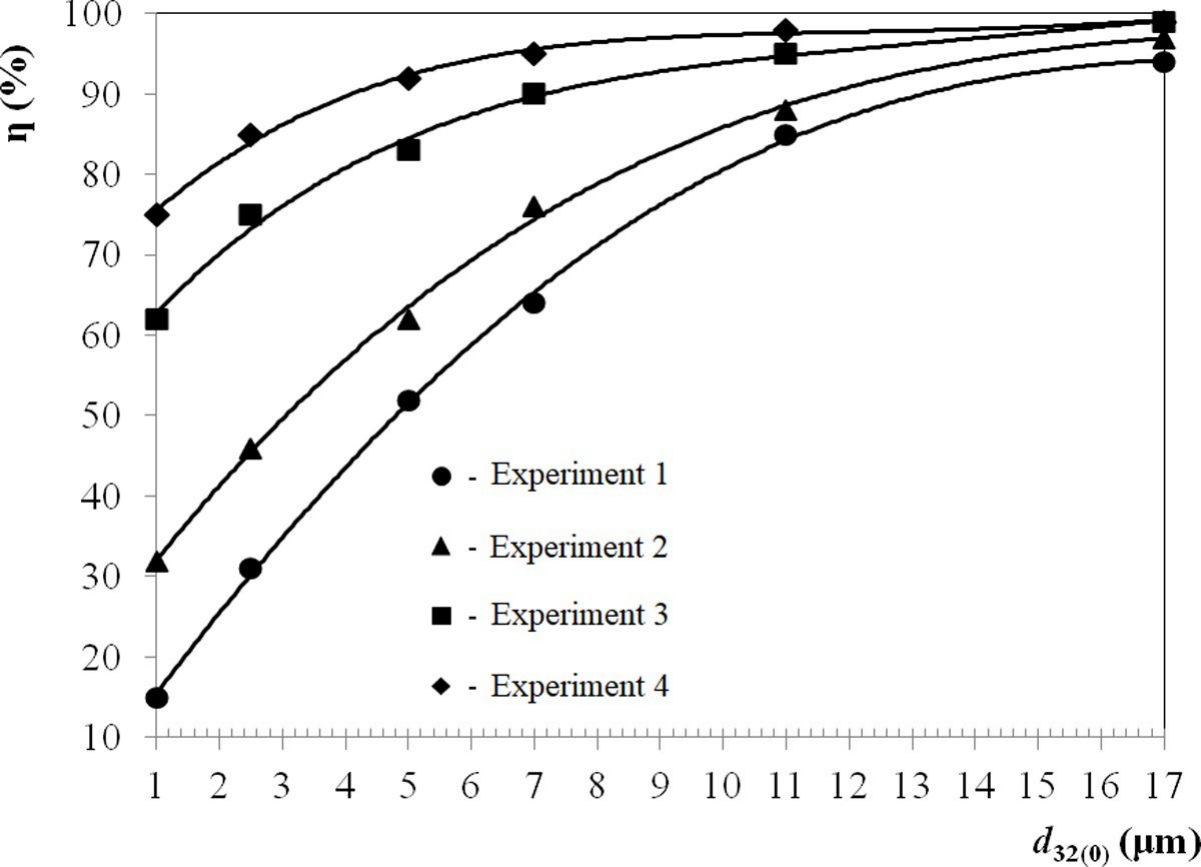
Fractional efficiency of the ultrasonic exposure in a swirling flow.

The obtained data on the fractional efficiency of trapping particles with and without ultrasound exposure are shown in Fig 9.

An analysis of the presented data shows that the swirling flow in the agglomerator (experiment 2 in Fig 9) improved the cleaning efficiency by 15% (from 31% to 46%) compared to a cyclone (a check experiment). This is due to the fact that in the agglomerator (even without ultrasonic vibrations) the processes of particle combining take place. Agglomeration occurs due to the diffusion of particles along the radius of the flow from the center to the periphery (the diffusion rate depends on the particle size; larger particles move faster, which increases the likelihood of their collision with small particles) and the effects of Brownian and turbulent coagulation [32].

Hereinafter, data are provided for particles with *d*_32_ = 2.5 μm. The ultrasonic exposure of the swirling flow in the agglomerator (experiment 3 in Fig 9) resulted in a significant increase in the gas cleaning efficiency of up to 75%.

In the last experiment, ultrasonic exposure was implemented in the agglomerator and cyclone, which ensured that the maximum cleaning efficiency of dispersed particles was 85%. The efficiency is higher for larger particles. The remaining agglomerates of dispersed particles (due to the enlarged size) can be easily captured by conventional gas cleaning equipment.

For comparison, according to the available experimental data, the cleaning efficiency for particles with *d*_32_ = 2.5 μm in the inertial gas cleaning equipment is within 20–50% [43].

Thus, the obtained results confirm the effectiveness of the proposed approach to increase the efficiency of ultrasonic agglomeration by forming local areas with an increased concentration of dispersed particles due to a swirling flow of dispersed particles.

## Conclusions

An approach to increasing the efficiency of ultrasonic agglomeration of dispersed particles less than 2.5 μm by converging the particles and forming local areas with high concentration was investigated. It is demonstrated that this is technically the easiest method to form such areas in a swirling flow.

The experimental equipment was developed, and studies were carried out to identify the optimal conditions for the formation of a swirling flow, which ensures an up to 4.5 times increase in average particle size of *d*_32_ *=* 2.5 μm. Thus, the cleaning efficiency of *d*_32_ *=* 2.5 μm particles can increase from 46% to 85%. The remaining agglomerates of dispersed particles (due to the increased size) can be easily captured by conventional gas cleaning equipment.

## Supporting information

S1 Data (XLSX)
